# Prevalence and risk factors of acne scars in patients with acne vulgaris

**DOI:** 10.1111/srt.13386

**Published:** 2023-06-05

**Authors:** Lin Liu, Yuzhou Xue, Yangmei Chen, Tingqiao Chen, Judan Zhong, Xinyi Shao, Jin Chen

**Affiliations:** ^1^ Department of Dermatology The First Affiliated Hospital of Chongqing Medical University Chongqing China; ^2^ Department of Cardiology and Institute of Vascular Medicine Peking University Third Hospital Beijing China

**Keywords:** acne, acne scar, acne vulgaris, prevalence, risk factor

## Abstract

**Background:**

Acne scar is a persistent complication of acne vulgaris. However, the prevalence and risk factors are still unclear. This study aimed to assess the global prevalence and risk factors of acne scars in patients with acne.

**Materials and methods:**

A systematic search of published studies in three databases was performed and the meta‐analyses were conducted.

**Results:**

Finally, we included 37 studies involving 24 649 acne patients. And, the pooled prevalence of acne scars in these patients was 47% (95% confidence interval [CI]: 38–56%). Besides, the differences in prevalence were observed based on the subgroup analysis for age, gender, acne severity, source of patients, and so on. Subsequently, we quantified the relationship of three risk factors with acne scars: male gender (odds ratio [OR]: 1.58, 95% CI: 1.19–2.09), positive family history of acne (OR: 2.73, 95% CI: 1.26–5.91), and acne severity (OR for moderate acne: 2.34, 95% CI: 1.54–3.57; OR for severe acne: 5.51, 95% CI: 2.45–12.41).

**Conclusion:**

Herein, we found that 47% of acne patients suffered from acne scars and identified three risk factors: male gender, positive family history of acne, and acne severity. In order to reduce acne scarring, attention and effective therapy early in the course of acne is important.

## 1 INTRODUCTION

Acne vulgaris is a common chronic inflammatory skin disorder and the eighth most prevalent disease worldwide, with a prevalence of 9.4%.^1^ Acne scar, one of the most persistent complications of acne, causes distress to the appearance and psychology of the patient.^2^ The pathogenesis of acne scars is unclear, but some studies suggest that it may involve changes in inflammation and fiber.^3^, ^4^ The process of acne scar formation can be broadly divided into two stages: increased tissue formation and loss or damage of tissue, corresponding to keloid or hypertrophic scar and atrophic scar, respectively.^5^ The atrophic scars include three subtypes: icepick or V‐shaped, rolling or M‐shaped, and boxcar or U‐shaped scars. Currently, although there are various treatments for acne scars, such as laser resurfacing, micro‐needling, chemical peels, and volumizing fillers, acne scars remain difficult to treat completely.

Due to their negative impact and intractability, acne scars have received increasing attention. Although several observational studies have reported the epidemiological data of acne scars, these studies show considerable variability.^6^, ^7^, ^8^ The global prevalence of acne scars in patients with acne remains unknown, and no meta‐analysis has yet been conducted for this problem. In addition, the risk factors of acne scars were not thoroughly described. Some risk factors were reported including the worst‐ever severity of acne, duration of acne, family history of atrophic acne scars, and lesion manipulation behaviors.^9^ However, the relationship between risk factors and acne scars were not quantified. Therefore, through a systematic and comprehensive literature search, we performed a meta‐analysis designed to assess the prevalence and risk factors of acne scars in patients with acne.

## 2 METHODS

This meta‐analysis was performed in accordance with the Preferred Reporting Items for Systematic Reviews and Meta‐Analyses guidelines.

### 2.1 Search strategy

Two investigators independently searched the PubMed, Web of Science, and EMBASE databases for studies published before January 09, 2023. The literature search was done using the following search terms: “acne AND (scar odds ratio [OR] scars OR scarring OR cicatrix OR cicatrization)” without any other limitation. To search for the relevant studies more comprehensively, additional articles were manually searched by checking reference lists of articles that included full‐text review.

### 2.2 Study selection and eligibility criteria

**FIGURE 1:**
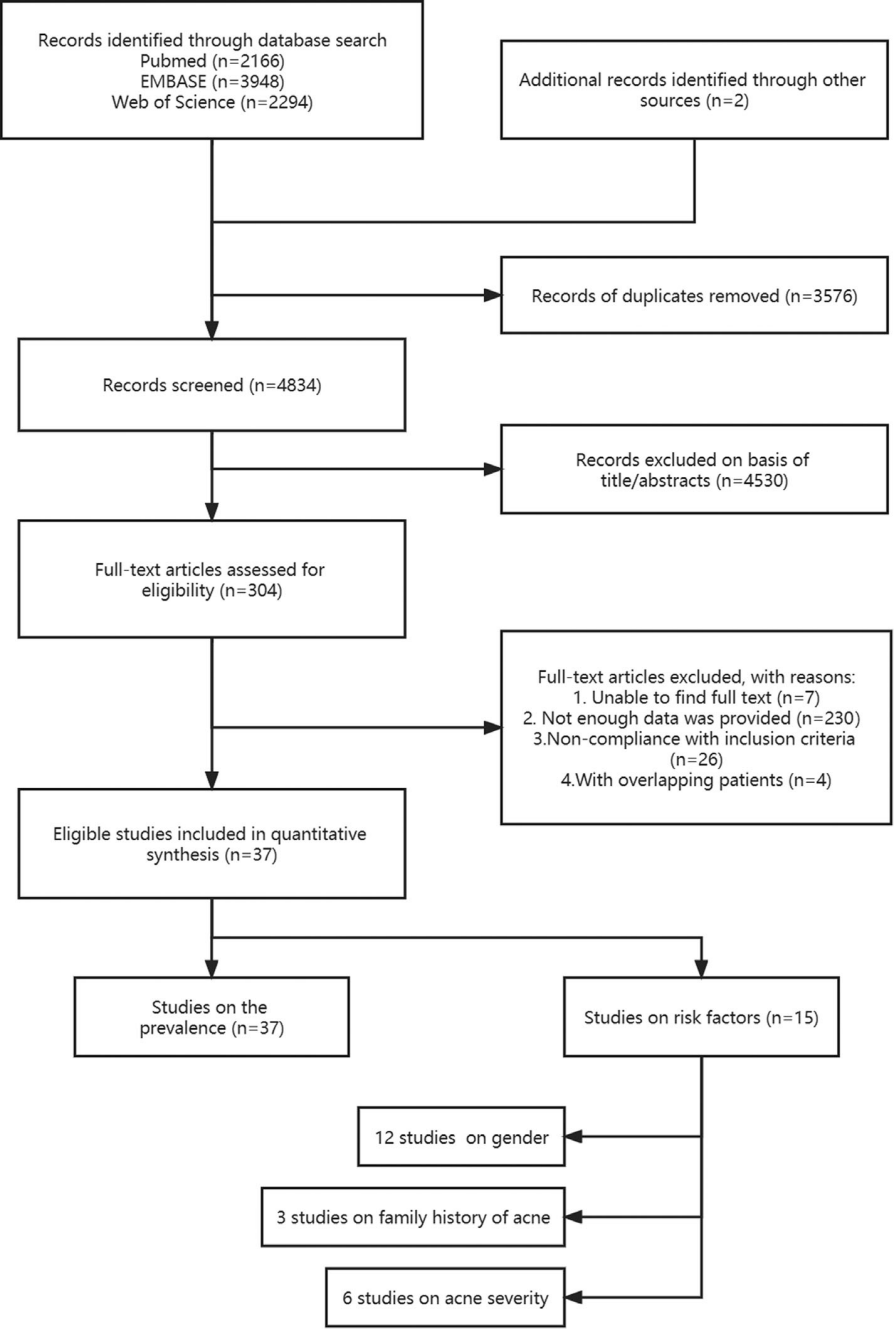
Flow‐chart of the selection process for the studies included in the meta‐analysis.

Firstly, we removed all records of duplicates. Then, the titles and abstracts of studies were independently examined by two investigators for the initial selection stage. Next, the second selection stage was based on a full‐text review by two investigators. The whole selection process was shown in Figure 1.

As for the eligibility criteria, studies were included if they met the following criteria: (1) original observational studies; (2) studies providing enough data to estimate the prevalence of acne scars in patients with acne and the corresponding 95% confidence interval (CI) and studies providing related date on odds ratio (OR) of risk factors; and (3) studies published in English. Further, if the analyzed patients of several studies came from the same population, the most informative study was chosen. Any differences of opinion or ambiguities that appeared during the examination were brought to the attention of a third senior review author.

### 2.3 Data extraction and quality assessment

For all eligible studies, two investigators extracted and tabulated data from them, including the following data: author, year, country, study design, definition of acne scar, number of patients, gender balance, age, source of patients, and assessment methods. Besides, for the quality assessment, the Newcastle–Ottawa Scale (NOS) was used to assess the quality of the eligible studies and an adapted version of NOS was applied for studies without controls.^10^

### 2.4 Data analysis

The aim of this study was to determine the prevalence and risk factors of acne scars in patients with acne. Thus, we performed meta‐analyses to obtain the pooled prevalence with 95% CIs and the ORs of risk factors with 95% CIs as main results.

Heterogeneity among the studies was examined using the *I* ^2^ statistic: low heterogeneity, *I* ^2^ < 25%; moderate heterogeneity, *I* ^2^ = 25%−50%, and high heterogeneity, *I* ^2^ > 50%. If *I* ^2^ was <50%, the fixed effect model was chosen; otherwise, the random effects model was chosen. However, considering the publication bias, all subgroup analyses used the random effect model based on the Der‐Simonian and Laird method.

And sensitivity analysis was conducted to evaluate the quality stability of the pooled results by omitting each study one at a time. In addition, publication bias was assessed using Egger's test and Begg's test. If the two‐sided values were <0.05, the results were considered to be statistically significant. All meta‐analyses were conducted using Stata version 12.0.

## 3 RESULTS

### 3.1 Search history and study characteristics

In the original search, a total of 7523 studies were identified from the PubMed, EMBASE, and Web of Science databases, together with additional sources, using the primary search strategy. In updated search, 887 studies were identified. A total of 4834 nonduplicate records were screened by titles and abstracts, yielding 304 studies for full‐text assessment. Finally, 37 studies were selected for this meta‐analysis, while the remaining studies were excluded for various reasons. The detailed search history is presented in Figure 1.

This meta‐analysis included 24 649 patients with acne from five continents in 37 studies published between 1992 and 2022. Among these 37 studies, the participants of nine nine studies were from the community (10 529 patients with acne), while the rest were from the clinic (14 120 patients with acne). Besides, 17 studies provided specific information about the prevalence in terms of sex distribution, including five studies that involved only female patients and one study that involved only male patients. Most of the study population included people of all ages, but three studies focused on adolescent acne and five focused on adult acne.

As for risk factors, the meta‐analysis of ORs on acne severity was conducted based on six studies, and three studies were included in meta‐analysis on family history of acne, while 12 studies on the ORs of gender.

Moreover, according to the NOS grading system, the quality of all studies with a score ≥7 was evaluated as high. More details about the characteristics of the study populations are shown in Table S1. No publication bias was found according to Egger's test (*p* > 0.05) and Begg's test (*p* > 0.05).

### 3.2 Prevalence of acne scars in patients with acne

**FIGURE 2:**
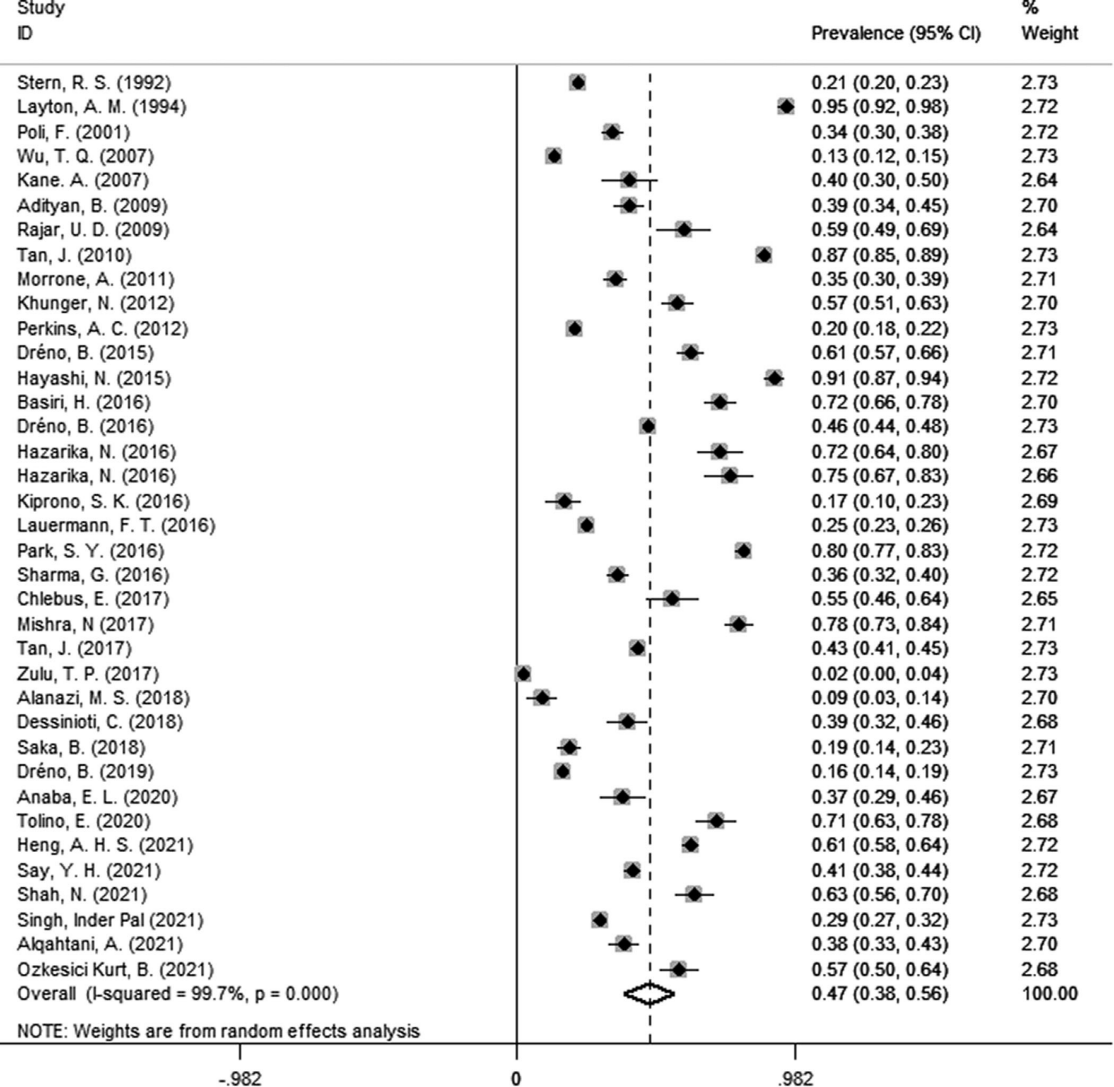
Forest plots of meta‐analysis for the pooled prevalence of acne scars in patients with acne. CI, confidence interval.

Overall, quantitative analysis of 37 studies yielded a pooled acne scar prevalence of 47% (95% CI: 38−56%) in patients with acne (Figure 2). Considering the heterogeneity was high (*I* ^2^ = 99.7%), we further processed sensitivity analysis and subgroup analysis. The result for sensitivity analysis confirmed the robustness of the pooled value (Figure S1). Besides, we did not find the source of high heterogeneity by subgroup analyses listed in Table 1. Additionally, no publication bias was found according to the Egger's test (*p* > 0.05) and Begg's test (*p* > 0.05).

**TABLE 1:** Subgroup analysis of the prevalence of acne scars in patients with acne.

Group	Studies	Prevalence	95% CI	Heterogeneity, *I* ^2^
Subtypes^†^				
Atrophic	8	0.78	69%–87%	0.965
Hypertrophic	8	0.17	11%–23%	0.938
Keloids	7	0.03	1%–5%	0.766
Ice‐pick	6	0.52	33%–71%	0.978
Rolling	6	0.24	12%–36%	0.958
Boxcar	4	0.25	8%–41%	0.960
Location^†^				
Cheek	3	0.68	52%–83%	0.972
Mandibular area	3	0.29	0%–59%	0.994
Forehead	3	0.35	22%–48%	0.995
Temple	2	0.29	14%–45%	0.951
Sex				
Female	17	0.46	34%–58%	0.995
Male	14	0.58	44%–72%	0.994
Age				
≥25 years	6	0.59	42%–75%	0.983
<25 years	7	0.32	18%–47%	0.995
Region				
Africa	6	0.31	11%–51%	0.989
Asia	19	0.52	39%–65%	0.995
Europe	7	0.51	29%–73%	0.996
North America	3	0.50	12%–89%	0.999
South America	2	0.20	10%–30%	0.935
Family history of acne				
Yes	2	0.49	47%–51%	0.000
No	2	0.40	0.35–0.45	0.195
Acne severity				
Mild acne	6	0.46	30%–62%	0.980
Moderate acne	6	0.67	34%–100%	0.998
Severe acne	6	0.82	64%–100%	0.989
Source of patients				
Community	9	0.27	18%–36%	0.992
Clinic	28	0.53	42%–65%	0.996
Assessment				
Physician‐diagnosed	32	0.48	38%–57%	0.997
Self‐report	6	0.47	21%–72%	0.997
Sample size				
*n* ≥ 500	14	0.40	27%–52%	0.998
*n* < 500	23	0.51	36%–67%	0.997

Abbreviation: CI, confidence interval.

^†^ Prevalence of subtype and location is the proportion conducted in patients with acne scar rather than patients with acne.

When analyzing the subtypes of acne scars, markedly different pooled proportions were found as follows: atrophic scars, 78% (95% CI: 69%−87%); hypertrophic scars, 17% (95% CI: 11%−23%); and keloids, 3% (95% CI: 1%−5%). Further, in the atrophic scars, icepick scars accounted for 52% (95% CI: 33%−71%); rolling scars, 24% (95% CI: 12%−36%); and boxcar scars, 25% (95% CI: 8%−41%). The differences in prevalence were also observed in the location of acne scars: 68% for cheek (95% CI: 52%−83%), 29% for mandibular area (95% CI: 0%−59%), 35% for forehead (95% CI: 22%−48%), and 29% for temple (95% CI: 14%−45%).

Stratified by sex, 17 studies provided specific data. A total of 8923 women and 5741 men were included, and the pooled prevalence in female patients was 46% (95% CI: 34%−58%), while that in male patients was 58% (95% CI: 44%−72%). Using the relative data of 10 studies, we divided the patients into two groups based on their ages: adult acne group (≥25 years) and nonadult acne group (<25 years). The prevalence of acne scars was 59% (95% CI: 42%−75%) in patients aged ≥25 years and 32% (95% CI: 18%−47%) in those aged <25 years. The pooled estimates for prevalence varied by region: 31% (95% CI: 11%−51%) in Africa, 52% (95% CI: 39%−65%) in Asia, 51% (95% CI: 29%−73%) in Europe, 50% (95% CI: 12%−89%) in North America, and 20% (95% CI: 10%−30%) in South America.

Based on data from studies that provided detailed information of patients with mild‐to‐severe acne, the prevalence of acne scars appeared to increase with severity: the proportion was 46% (95% CI: 30%−62%) in mild acne, 67% (95% CI: 34%−100%) in moderate acne, and 82% (95% CI: 64%−100%) in severe acne. The pooled prevalence of acne was 27% (95% CI: 18%−36%) in community patients and 53% (95% CI: 42%−65%) in clinic patients. The prevalence was 48% (95% CI: 38%−57%) in studies assessing physician‐diagnosed cases and 47% (95% CI: 21%−72%) in studies assessing self‐reported cases. Studies involving ≥500 patients yielded a prevalence of 40% (95% CI: 27%−52%), whereas those involving <500 patients yielded a prevalence of 51% (95% CI: 36%−67%). The results for subgroup analysis are shown in Table 1.

### 3.3 Pooled ORs of risk factors in acne scars

**FIGURE 3:**
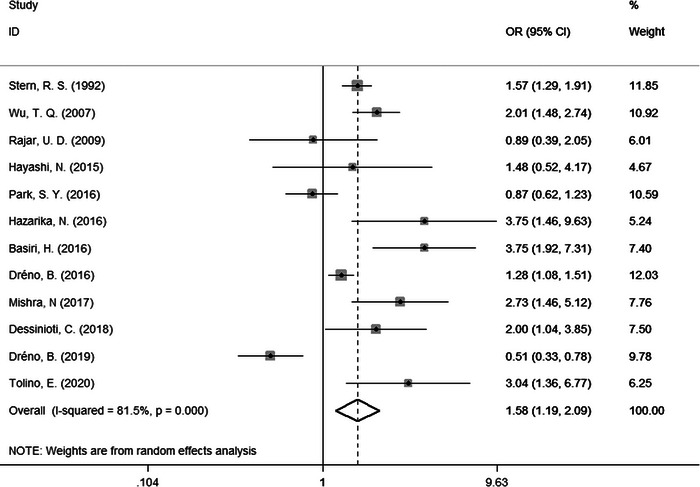
Forest plots of meta‐analysis for the OR of acne scars by sex. CI, confidence interval; OR, odds ratio.

**FIGURE 4:**
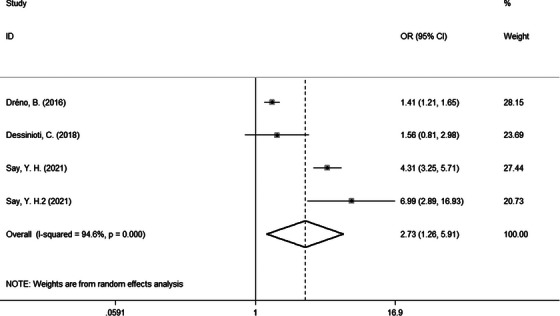
Forest plots of meta‐analysis for the OR of acne scars by family history of acne. CI, confidence interval; OR, odds ratio.

**FIGURE 5:**
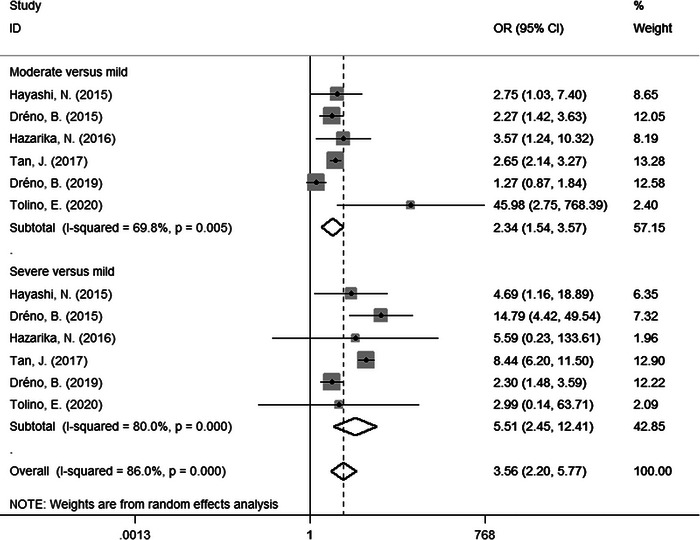
Forest plots of meta‐analysis for the OR of acne scars by acne severity. CI, confidence interval; OR, odds ratio.

From the subgroup analysis, we noted that some factors might be related to acne scars. Therefore, we further performed meta‐analysis on ORs for risk factors. Finally, we found three risk factors. Compared to female patients, male patients were more likely to suffer from acne scars (OR = 1.58, 95% CI: 1.19−2.09, Figure 3). Besides, positive family history of acne was also related to acne scars (OR = 2.73, 95% CI: 1.26−5.91, Figure 4). Moreover, there were significant differences in moderate versus mild acne (OR = 2.34, 95% CI: 1.54−3.57) and severe versus mild acne (OR = 5.51, 95% CI: 2.45−12.41) (Figure 5). There were no significantly different results in the Egger's test (*p* > 0.05), Begg's test (*p* > 0.05), or sensitivity analysis (Figure S2).

## 4 DISCUSSION

In this meta‐analysis, the prevalence of acne scars among patients with acne was 47%. This pooled value was similar to previous studies on large population.^11^, ^12^ Although we observed high heterogeneity, the source of heterogeneity did not appear to be identified through subgroup analysis and sensitivity analysis. The heterogeneity decreased in subgroup analysis on family history of acne; however, it included only two studies and the result was not persuasive. In subgroup analyses, the differences in prevalence were observed in the subtype and location of acne scars, sex, age, region, family history of acne, acne severity, source of patients, assessment and sample size. Later, our further analysis found three risk factors: male gender, positive family history of acne, and acne severity. In fact, we also concerned the roles of other factors, such as duration of acne, lifestyle, squeezing behaviors, and the relapse of acne. But due to lack of sufficient data, it could not be analyzed.

We found that positive family history of acne increased the risk of acne scars (OR = 2.73, 95% CI: 1.26−5.91). Acne had a highly heritable trait and some genetic susceptibility loci, including *SELL* (i.e., Selectin L) and *TGFB2* (i.e., Transforming Growth Factor Beta 2), which were implicated in scarring, were identified.^13^ Genetic factors might play a role in acne scarring. For innate immunity profiles, the differences between patients prone to scars (PS) and not prone to scars (NPS) were observed even in normal skin.^14^ Besides, Type IV delayed hypersensitivity response was found in NPS, while a predominantly adaptive immune response was present in PS.^15^ And inflammatory immune processes persisted longer in PS and plasma cells are specifically involved in immune response of evolved‐lesions.^16^ These clues suggested that there might be a close connection between acne scars and genetic factors, but more studies were needed for further exploration.

In the subgroup meta‐analysis, we found that the estimates for prevalence in mild, moderate, and severe acne were 46%, 67%, and 82%, respectively. Our further meta‐analysis suggested that acne severity was a risk factor for acne scars, with an OR of 2.34 (95% CI: 1.54−3.57) in moderate versus mild acne and 5.51 (95% CI: 2.45−12.41) in severe versus mild acne. Our results showed that acne scars affected all levels of acne, and that the prevalence of acne scars increased with the severity of acne. Severe acne was often accompanied by prolonged and exacerbated skin inflammation, which was more likely to result in acne scars. However, it was notable that the prevalence of acne scars in mild acne was as high as 46% in our study. We were supposed to recognize that acne was a disease of sebaceous gland unit,^17^ and long‐term inflammatory response could cause irreversible destruction of sebaceous gland structure, contributing to atrophic scar formation.^16^ Although the inflammation in mild acne was limited to some units and the inflammation degree was milder than that in severe acne,^18^ the destruction of these units could also cause acne scars. Therefore, it was a mistake that only patients with severe acne were examined and treated for acne scars. It is also important to treat mild acne to prevent development of acne and formation of scars.

Previous studies have reported that acne scars were more common in men than in women.^19^ Our study yielded a prevalence of 58% in men and 46% in women, and further analysis indicated that male gender might be a risk factor for acne scars (OR = 1.58, 95% CI: 1.19−2.09). Men were more likely to suffer from severe acne than women,^20^ which might be associated with high androgen level^21^ and special sebaceous gland.^22^ Men were found to have cauliflower‐shaped sebaceous glands and depth‐dependent differences in sebaceous unit areas,^22^ and this special shape might lead men to be more prone to severe acne than women, contributing to acne scar formation. Another possible reason was that most women were more self‐conscious of their appearance than men and were more likely to seek help from their physicians for acne. As a result of the early and timely treatment, they had a lower chance of developing acne scars. The above factors might contribute to gender differences in acne scarring, but the details needed to be further studied.

We also found that the prevalence of acne scars in the adult acne group (59%) was significantly higher than that in the nonadult acne group (32%). The adult acne was usually mild or moderate in severity. However, since most of the lesions were inflammatory, they might become resistant to drugs such as antibiotics and isotretinoin,^23^ leading to greater scarring. However, it must be noted that in the adult acne group, the prevalence of acne scars in late‐onset and persistent acne was unknown. It was also unclear whether the process of scar formation differed between late‐onset and persistent acne. These problems require further investigation to explore their distinctions.

The prevalence of acne scars also showed geographical differences. In comparison with Asia (52%) and Europe (51%), we observed a lower prevalence in Africa (31%), where the majority of the population was dark‐skinned. Interestingly, individuals with dark skin were more prone to keloid and hypertrophic scars.^24^ However, they were less likely to develop acne scars, which were predominantly of the atrophic type. This might be related to genetic susceptibility, including major histocompatibility complex genes and *SMAD* (i.e., SMAD Family Member 2) genes,^25^, ^26^ but the exact mechanism needs to be determined. Moreover, in people with dark skin, small atrophic scars might be ignored during visual examination.

This study had some limitations. First, as mentioned above, there was high heterogeneity for the pooled prevalence. Second, small sample size of some included studies might result in unsatisfactory representativity. Third, in terms of source of patients, the prevalence was much higher in clinic patients (53%) than in community patients (27%). Individuals with more severe acne and embarrassing scars tended to seek medical advice more frequently. Thus, a proportion of patients with mild acne and mini acne scars who did not visit the hospital might have been missed. Because most of our included studies were performed in the clinic, the pooled prevalence might have been overestimated. In addition, the measure of assessment was also different: some were by physician‐diagnosed, while others were by self‐reported. Lack of unified measurement standards could lead to significant differences in results. Therefore, further well‐designed large prospective studies, taking into account these potential confounders, are required in the future.

In conclusion, based on the published data, this meta‐analysis found that 47% of patients with acne suffered from acne scars and male gender, positive family history of acne, and acne severity were risk factors for acne scars. In order to prevent further aggravation of acne and formation of scars, it is important to treat acne at an early stage.

## CONFLICT OF INTEREST STATEMENT

All authors declare no potential conflict of interest.

## ETHICS STATEMENT

This study did not require ethical approval as the data used have been published previously and hence are already in the public domain.

## Supporting information

Supporting Information

Supporting Information

## Data Availability

The data that support the findings of this study are available from the corresponding author upon reasonable request.
