# Supplementary material for: Prevalence and risk factors of acne scars in patients with acne vulgaris
Source: Skin Res Technol. 2023 Jun 5;29(6):e13386. doi: 10.1111/srt.13386 (PMC10240192; doi:10.1111/srt.13386)
Supplement: Supplementary file 1 — Supporting Information [file SRT-29-e13386-s002.docx]

**Supporting Information**


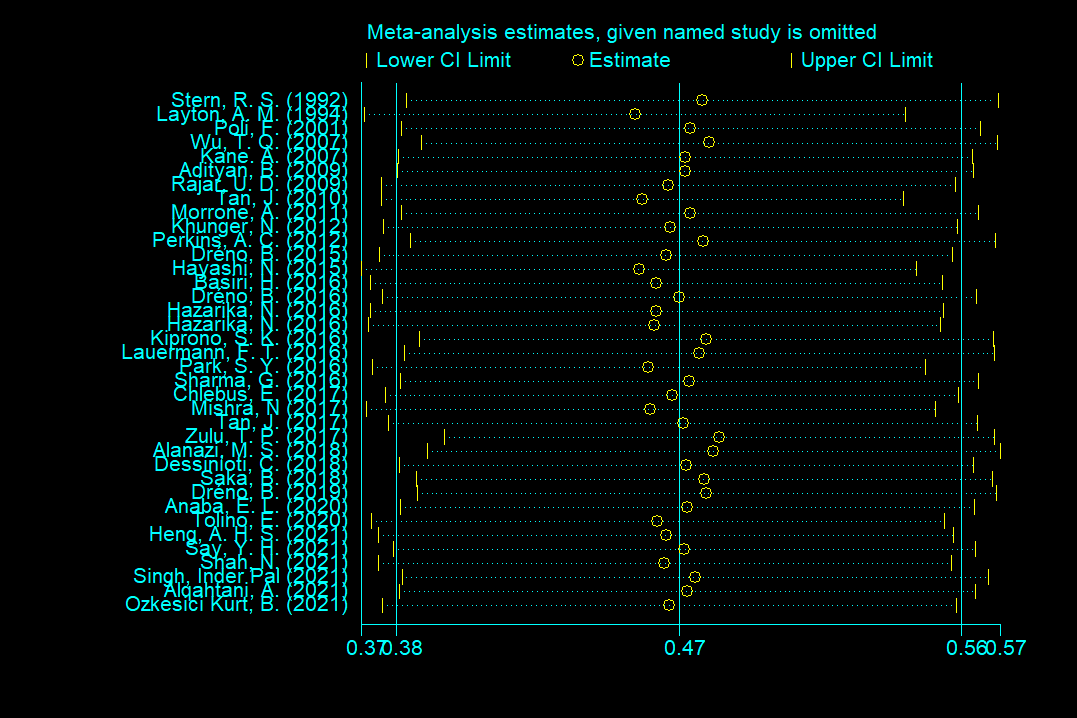


Figure S1: Sensitivity analysis of meta-analysis for prevalence.


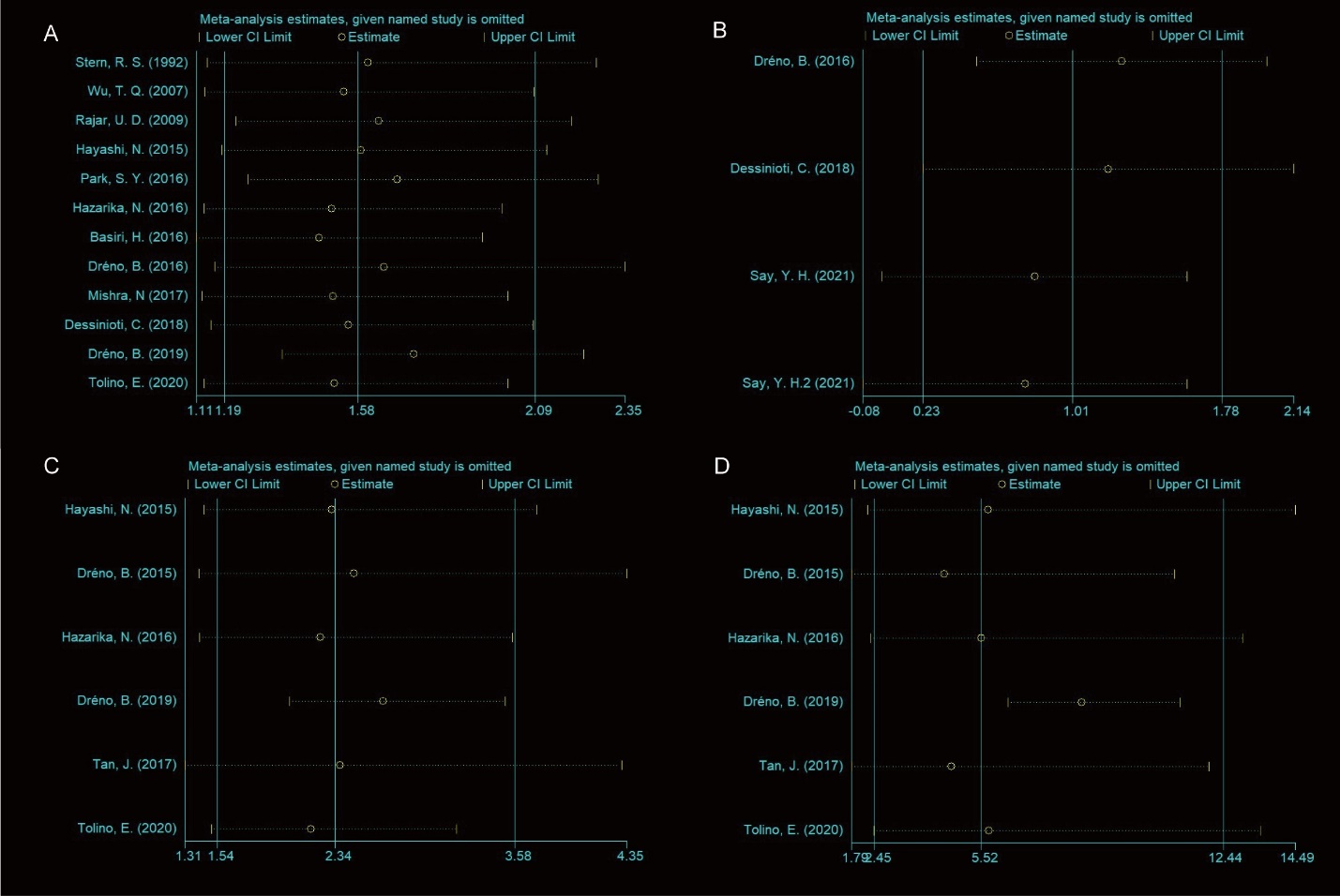


Figure S2: Sensitivity analysis of meta-analysis for risk factors: (A) sex; (B) family history of acne; (C) moderate versus mild acne; (D) severe versus mild acne.
