# Supplementary material for: Prevalence and risk factors of acne scars in patients with acne vulgaris
Source: Skin Res Technol. 2023 Jun 5;29(6):e13386. doi: 10.1111/srt.13386 (PMC10240192; doi:10.1111/srt.13386)
Supplement: Supplementary file 2 — Supporting Information [file SRT-29-e13386-s001.docx]

**Table S1.** **Characteristics of studies included in the meta-analysis and Newcastle–Ottawa Scale (NOS) quality assessment**

| Author | Year | country | Study design | Definition of acne scar | Sample Size  (event/total) | Sex  (Male/Female) | Mean age/ age range | Source of patients | Assessment | NOS score |
| --- | --- | --- | --- | --- | --- | --- | --- | --- | --- | --- |
| Stern, R. S. et al | 1992 | USA | Cross-sectional study | One or more pitted scars ("ice-pick" scars); Cystic scars | 521/2426 | 925: 1501 | NA/15-44 | Stratified random sample of residents in 1970-1974 | Physician-diagnosed | 9 |
| Layton, A. M. et al | 1994 | UK | Observational study | Ice-pick, macular atrophic and follicular macular atrophic scars | 176/185 | 84: 101 | NA | Clinic patients | Physician-diagnosed | 8 |
| Poli, F. et al | 2001 | France | Cross-sectional study | Atrophic scars | 195/575 | 0: 575 | NA/25-40 | Household | Self-report | 7 |
| Wu, T. Q. et al | 2007 | China | Cross-sectional study | Ice pick scars (pitted scars) and atrophic scars (flat, thin scars) | 224/1691 | 982: 7095 | NA/10-18 | Adolescents from public schools | Physician-diagnosed | 9 |
| Kane. A. et al | 2007 | Africa | Cross-sectional study | Scars | 37/93 | 23:70 | 25.58/14-46 | Clinic patients in 2002.12-2003.03 | Self-report | 7 |
| Adityan, B. et al | 2009 | India | Cross-sectional study | Nondistensible deep (ice-pick); Distensible retraction; Distensible undulation; Nondistensible superficial (dish-like); Nondistensible medium (crater-like); Hypertrophic scar; Keloid | 122/309 | 172: 137 | 19.78±4.94/13-45 | Clinic patients in 2006.08-2008.06 | Physician-diagnosed | 7 |
| Rajar, U. D. et al | 2009 | India | Cross-sectional study | Ice-pick scars; Box scars; Rolling scars; Keloidal scars; Hypertrophic scars | 59/100 | 35: 65 | 18.70±4.50/11-35 | Clinic patients in 2007.09-2008.02 | Physician-diagnosed | 8 |
| Tan, J. et al | 2010 | Canada | Cross-sectional study | Scars | By self-report: 710/973  By Physician-diagnosed:  847//973 | 409: 564 | 25.4±7.9/13-75 | Clinic patients | Physician-diagnosed;  Self-report | 9 |
| Morrone, A. et al | 2011 | Italy | Cross-sectional study | Scarring | 155/444 | 165: 279 | 19/14-35 | Clinic patients in 2005.01-2008.12 | Physician-diagnosed | 7 |
| Khunger, N. et al | 2012 | India | Cross-sectional study | Ice pick scars; Rolling scars; Keloidal scars | 159/280 | 50: 230 | 30.5/26-50 | Clinic patients in 2008.11-2010.04 | Physician-diagnosed | 8 |
| Perkins, A. C. et al | 2012 | England, Italy, Japan, USA | Cross-sectional study | Scarring | 325/1592 | 0: 1592 | NA/10-70 | General population. | Physician-diagnosed by photo | 8 |
| Hayashi, N. et al | 2015 | Japan | Cross-sectional study | "Mini-scars (atrophic scars of ≥0.5 and <2 mm in  diameter); Atrophic scars (≥2 mm in diameter); Hypertrophic  scars, including keloids." | 218/240 | 71: 169 | 25.0±6.7/NA | clinic patients | Physician-diagnosed | 8 |
| Dréno, B. et al | 2015 | 15 countries* | Cross-sectional study | Atrophic scars; Hypertrophic scars | 230/374 | 0: 374 | 32.0/25-66 | Clinic patients in 2011.01-2012.09 | Physician-diagnosed | 9 |
| Park, S. Y. et al | 2016 | Korea | Cross-sectional study | Scars | 720/900 | 293: 607 | 24.6±5.3/12-39 | Clinic patients in 2013.06-2013.07 | Self-report | 7 |
| Lauermann, F. T. et al | 2016 | Brazil | Cross-sectional study | Scars | 483/1968 | 1968: 0 | 18/NA | Adolescents in military service | Physician-diagnosed | 7 |
| Kiprono, S. K. et al | 2016 | Kenya | Cross-sectional study | Atrophic scars; Hypertrophic scars; Keloids | 22/133 | 0: 71 | 15/9-52 | General population of rural area | Physician-diagnosed | 9 |
| Hazarika, N. et al | 2016 | India | Cross-sectional study | Acne scars (all types included) | 82/114 | 49: 65 | 19.39/NA | Clinic patients in 2014.06-2014.11 | Physician-diagnosed | 8 |
| Hazarika, N. et al | 2016 | India | Cross-sectional study | Acne scars | 75/100 | 44: 56 | NA | Clinic patients in 2015.01-2015.03 | Physician-diagnosed | 7 |
| Sharma, G. et al | 2016 | India | Cross-sectional study | Post-acne scarring | 194/545 | 320: 225 | 20.4±4.2/12-43 | Clinic patients in 2015.06-2015.11 | Physician-diagnosed | 8 |
| Basiri, H. et al | 2016 | Iran | Cross-sectional study | Acne scars (all types included) | 164/228 | 98: 130 | 19.39/ age>15 | Clinic patients in 2015.12-2016.01 | Physician-diagnosed | 8 |
| Dréno, B. et al | 2016 | France, Switzerland, Italy, Portugal | Cross-sectional study | V-shaped scar; U-shaped scar; M-shaped scar; Superficial elastolysis; Inflammatory hypertrophic scars; Hypertrophic cheloid scars | 1295/2797 | 752: 2045 | 22.5±8.0/4-66 | Clinic patients from 2010.08-2011.11 | Physician-diagnosed | 7 |
| Chlebus, E. et al | 2017 | Poland | Cohort study | Scars | 61/111 | 10: 101 | NA/25-50 | Clinic patients in 2015.05-2016.01 | Physician-diagnosed | 9 |
| Zulu, T. P. et al | 2017 | The Republic of South Africa | Cross-sectional study | Keloids and scarring | 5/242 | 38: 204 | 28.5/18-59 | Clinic patients in 2014.01-2014.12 | Physician-diagnosed | 8 |
| Mishra, N et al | 2017 | India | Cross-sectional study | Significant scarring | 196/250 | 178: 72 | 19.89±3.42/age>14 | Clinic patients in 2012.04-2013.03 | Physician-diagnosed | 7 |
| Tan, J. et al | 2017 | USA | Cohort study | Ice pick (0.5 to <2 mm); Atrophic acne scars 2-4 mm; Atrophic acne scars >4 mm; Hypertrophic or keloidal scars | 843/1972 | 828: 1144 | 22.9/9-72 | Clinic patients in 2012.05-2013.03 | Physician-diagnosed | 8 |
| Alanazi, M. S. et al | 2018 | Saudi Arabia | Cross-sectional study | Scarring | 9/104 | 0: 104 | NA/14-21 | Female students in secondary school | Physician-diagnosed | 9 |
| Dessinioti, C. et al | 2018 | Greece | Cross-sectional study | Scarring | 65/167 | 58: 109 | 20.7±5.68/NA | Clinic patients | Physician-diagnosed | 8 |
| Saka, B. et al | 2018 | Togo | Cross-sectional study | Atrophic scars; Hypertrophic scars | 56/300 | 120: 180 | 23.7±5.7/12-55 | clinic patients in 2017.07-2018.02 | Physician-diagnosed | 9 |
| Dréno, B. et al | 2019 | France | Cross-sectional study | Acne leave scars | 172/1048 | 278: 770 | NA/age>15 | Clinic patients in 2017.7-2018.3 | Self-report | 7 |
| Anaba, E. L. et al | 2020 | Nigeria | Cross-sectional study | Post inflammatory hyperpigmentation; Ice pick scars; Box scars, Keloid. | 50/134 | NA | NA/age>18 | Clinic patients in 2018.02-2019.01 | Physician-diagnosed | 8 |
| Tolino, E. et al | 2020 | Italy | Cross-sectional study | Hypertrophic scars; Atrophic scars | 106/150 | 60: 90 | NA/12-36 | clinic patients in 2017 | Physician-diagnosed | 8 |
| Shah, N. et al | 2021 | India | Cross-sectional study | Ice-pick scars; Boxcar scars; Rolling scars; Keloidal scars | 113/180 | 33: 147 | 30.12±4.83/26-48 | clinic patients | Physician-diagnosed | 8 |
| Say, Y. H. et al | 2021 | Malaysia | Cross-sectional study | Acne scarring | 431/1052 | 352: 700 | 21±4.926/17-77 | Students and staff in university | Physician-diagnosed | 8 |
| Heng, A. H. S. et al | 2021 | Singapore | Cross-sectional study | Scarring | 606/988 | 442: 546 | 21±4.6/17-71 | Students and staff in university | Physician-diagnosed | 8 |
| Singh, Inder Pal. et al | 2021 | India | Cross-sectional study | Acne scarring | 410/1392 | 834:558 | 21.15±5.16/18-25 | Clinic patients in 2017.1-2020.6 | Physician-diagnosed | 9 |
| Alqahtani, A. et al | 2021 | Saudi Arabia | Cross-sectional study | Scar | 114/300 | 67:233 | NA/15-30 | Clinic patients | Self-report | 7 |
| Özkesici Kurt, B. | 2021 | Turkey | Observational study | Scar | 110/192 | 60:132 | 19.42 ± 3.50/NA | Clinic patients | Physician-diagnosed | 8 |

*The 15 countries include: Denmark, Italy, France, Germany, Morocco, Russia, Spain, Argentina, Chile, USA, Australia, Japan, Korea, Philippines and Singapore.NA, not available.
